# Cell Death in Cyanobacteria: Current Understanding and Recommendations for a Consensus on Its Nomenclature

**DOI:** 10.3389/fmicb.2021.631654

**Published:** 2021-03-03

**Authors:** Anabella Aguilera, Marina Klemenčič, Daniela J. Sueldo, Piotr Rzymski, Leda Giannuzzi, María Victoria Martin

**Affiliations:** ^1^Centre for Ecology and Evolution in Microbial Model Systems (EEMiS), Linnaeus University, Kalmar, Sweden; ^2^Department of Chemistry and Biochemistry, Faculty of Chemistry and Chemical Technology, University of Ljubljana, Ljubljana, Slovenia; ^3^School of Life Sciences, University of Warwick, Coventry, United Kingdom; ^4^Department of Environmental Medicine, Poznan University of Medical Sciences, Poznan´, Poland; ^5^Integrated Science Association (ISA), Universal Scientific Education and Research Network (USERN), Poznan´, Poland; ^6^Centro de Investigación y Desarrollo en Criotecnología de Alimentos, Consejo Nacional de Investigaciones Científicas y Tecnológicas, Universidad Nacional de La Plata, La Plata, Argentina; ^7^Área de Toxicología General, Facultad de Ciencias Exactas, Universidad Nacional de La Plata, La Plata, Argentina; ^8^Instituto de Investigaciones en Biodiversidad y Biotecnología (INBIOTEC-CONICET), Fundación para Investigaciones Biológicas Aplicadas (CIB-FIBA), Mar del Plata, Argentina

**Keywords:** regulated cell death, caspases, cyanobacterial blooms, cyanophages, reactive oxygen species, apoptosis-like, ferroptosis-like

## Abstract

Cyanobacteria are globally widespread photosynthetic prokaryotes and are major contributors to global biogeochemical cycles. One of the most critical processes determining cyanobacterial eco-physiology is cellular death. Evidence supports the existence of controlled cellular demise in cyanobacteria, and various forms of cell death have been described as a response to biotic and abiotic stresses. However, cell death research in this phylogenetic group is a relatively young field and understanding of the underlying mechanisms and molecular machinery underpinning this fundamental process remains largely elusive. Furthermore, no systematic classification of modes of cell death has yet been established for cyanobacteria. In this work, we analyzed the state of knowledge in the field of cyanobacterial cell death. Based on that, we propose unified criterion for the definition of accidental, regulated, and programmed forms of cell death in cyanobacteria based on molecular, biochemical, and morphologic aspects following the directions of the Nomenclature Committee on Cell Death (NCCD). With this, we aim to provide a guide to standardize the nomenclature related to this topic in a precise and consistent manner, which will facilitate further ecological, evolutionary, and applied research in the field of cyanobacterial cell death.

## Introduction

Cyanobacteria are the only oxygenic photosynthetic prokaryotes, and prosper in diverse and extreme habitats (Whitton, 2012). They are among the oldest organisms on Earth with fossil records dating back 3.5 billion years (Schopf and Packer, 1987). Since then, cyanobacteria have been essential players in the Earth’s ecosystems. Planktonic cyanobacteria are a fundamental component of aquatic food webs and are major contributors to global carbon and nitrogen fluxes (Bullerjahn and Post, 2014; Tang et al., 2019). Moreover, some cyanobacteria form harmful algal blooms causing the disruption of aquatic ecosystem services and intoxication of wildlife and humans by the production of powerful toxins (cyanotoxins) such as microcystins, saxitoxin, and cylindrospermopsin (Bláha et al., 2009; Paerl and Otten, 2013). Nowadays, cyanobacterial blooms pose a serious threat to aquatic environments and public health, and are increasing in frequency and magnitude globally (Huisman et al., 2018).

Cyanobacteria present remarkable variability in terms of morphology: from unicellular and colonial to filamentous forms. Filamentous forms exhibit functional cell differentiation such as heterocysts (for nitrogen fixation), akinetes (resting stage cells), and hormogonia (reproductive, motile filaments). These, together with the intercellular connections they possess, are considered the first signs of multicellularity (Claessen et al., 2014; Nürnberg et al., 2014; Herrero et al., 2016).

Cell death is an important part of the microbial life-history that has been largely overlooked. However, reports on cell death of marine and freshwater cyanobacteria indicate that this process has major implications for the ecology of microbial communities (Agustí, 2004; Agustí et al., 2006; Franklin et al., 2006; Sigee et al., 2007). Different forms of cell demise have been observed in cyanobacteria under several stressful conditions (Berman-Frank et al., 2004; Hu and Rzymski, 2019), and cell death has been suggested to play a key role in developmental processes, such as akinete and heterocyst differentiation (Meeks et al., 2001; Claessen et al., 2014). Despite its ecological importance, the pathways that underpin the execution of cell death in cyanobacteria have been poorly characterized and remain largely unknown. Furthermore, the corresponding terminology to describe the process is heterogenous and potentially misleading.

**Figure 1:**
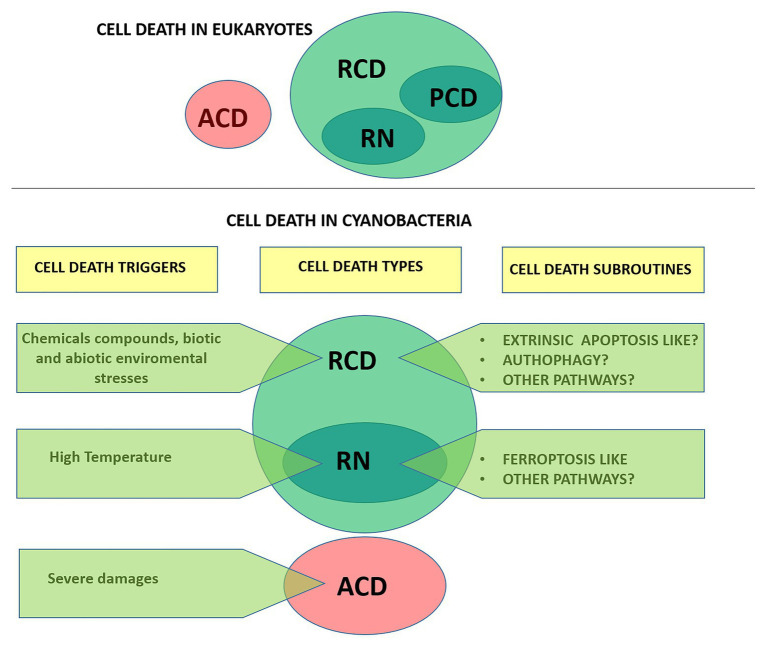
Types of cell death according to the Nomenclature Committee on Cell Death (**upper panel**; Galluzzi et al., 2015) and proposed for cyanobacteria **(lower panel)**. Cells exposed to extreme injury die in an uncontrollable manner, reflecting the loss of structural integrity. This type of cell death is called “accidental cell death” (ACD). “Regulated cell death (RCD)” is encoded by a genetic pathway that can be modulated by genetic or pharmacologic interventions. “Programmed cell death” (PCD) is a type of RCD that occurs as a developmental program, and has not been addressed in cyanobacteria yet. RN, regulated necrosis.

In animals, the current classification system of cell death is updated by the Nomenclature Committee on Cell Death (NCCD). Since 2005, the NCCD has established guidelines and recommendations, releasing five articles dealing with the classification of cell death (Kroemer et al., 2005, 2009), the molecular definitions of cell death subroutines (Galluzzi et al., 2012), essential vs. accessory aspects of cell death (Galluzzi et al., 2015), and the molecular mechanisms of cell death (Galluzzi et al., 2018). Currently, cell death is divided based on functional aspects into two main types: accidental cell death (ACD) and regulated cell death (RCD; Figure 1). ACD is an unpreventable and uncontrollable process caused by extreme physical, chemical or mechanical triggers. In contrast, RCD involves precise signaling cascades and relies on the intracellular molecular machinery, and can therefore be modulated pharmacologically or genetically (Galluzzi et al., 2015). In the absence of any exogenous environmental perturbation, RCD in animals plays a critical role in essential physiological programs such as embryonic development, differentiation, fertilization, and tissue renewal (Conradt, 2009). In this physiological context, RCD is generally referred to as programmed cell death (PCD). On the other hand, RCD can also occur in the context of adaptation to stress, when responses to perturbations of the intracellular or extracellular microenvironment fail, and therefore it would constitute an ultimate attempt to maintain cell homeostasis (Galluzzi et al., 2018). RCD is not unique to multicellular life forms. Several forms of RCD have been described in unicellular eukaryotes like yeast, kinetoplastid parasites (e.g., *Trypanosoma cruzi*), and algae (e.g., green algae, diatoms, haptophytes, dinoflagellates) (Ameisen, 2002; Bidle, 2016; Carmona-Gutierrez et al., 2018). In recent years, evidence that RCD also occurs in prokaryotes (Bayles, 2014; Allocati et al., 2015) including cyanobacteria (Bidle, 2016) has rapidly grown.

In this work, we review available information regarding cell death in cyanobacteria and discuss the terminology adopted in the field. Unless otherwise specified, it should be emphasized that we followed the exact terminology adopted by the authors in their studies and denoted it on single quotations marks. Furthermore, in an attempt to provide a formal characterization of cell death in cyanobacteria we follow the directions of the NCCD adapting them to this particular group (Figure 1).

Finally, in order to generate consensus, we provide a guide to standardize the nomenclature related to cell death in a precise and consistent way, which will need continuous revision.

Overall, the understanding of the mechanisms controlling cell demise in cyanobacteria will shed light on the under-explored field of RCD. In addition, it will motivate future experimental and field studies on this phenomenon, and open new applications in ecology, biotechnology, and management of water bodies with harmful cyanobacterial blooms.

## Nomenclature and Terms Adopted in the Literature to Refer to Cell Death in Cyanobacteria

Cyanobacterial cell death, as an active and genetically controlled process, has been traditionally considered analogous to PCD in multicellular organisms. Therefore, research in this group has followed the terminology and distinctions adopted for metazoans and plants (Hochman, 1997; Aravind et al., 1999; Berman-Frank et al., 2004; Franklin et al., 2006).

The concept of PCD as the active participation of the cell in its own death was introduced for the first time in plant cells in 1923 (Allen, 1923). PCD has been described as part of normal plant development, as well as a response to biotic and abiotic stresses (Jones, 2001; Huysmans et al., 2017). Despite research on the subroutines operating in plants has grown considerably in the past few years, their classification, and homology to animal system are still under debate (van Doorn et al., 2011; Huysmans et al., 2017; Bhattacharjee and Mishra, 2020).

The first classifications of cell death in animals were based purely on morphology, with limited reference to the underlying biochemical changes (Kroemer et al., 2005). Macroscopic morphological alterations observed in mammalian cells undergoing death were used to classify cell death in three main types: (i) apoptosis, exhibiting cytoplasmic shrinkage, chromatin condensation, nuclear fragmentation and plasma membrane bebbling and the formation of small vesicles or apoptotic bodies; (ii) autophagy, characterized by extensive cytoplasmic vacuolization; and (iii) necrosis, occurring without morphologic features of apoptosis or autophagy (Galluzzi et al., 2018). Of note, for a long time, necrosis was considered as an accidental non-RCD routine, or even as a strict equivalent of ACD (Galluzzi et al., 2015). However, regulated necrosis has been found to occur not only in animals (mammals and invertebrates) but also in plants and yeasts (Vandenabeele et al., 2010; Dixon et al., 2012; Distéfano et al., 2017; Carmona-Gutierrez et al., 2018). Since the morphological classification has multiple limitations and caveats, the NCCD proposes to define cell death subroutines on biochemical, genetic, pharmacological, and functional basis over morphology. Based on this classification, major subroutines operating in metazoans (mainly based on studies of mammalian cells) include: intrinsic and extrinsic apoptosis, autophagic cell death, necroptosis, and ferroptosis (Galluzzi et al., 2015, 2018).

In cyanobacteria, the term “proapoptosis” was early used to engage the manifestation of cells observed under stress, and it was postulated as the phylogenetic precursor of metazoan apoptosis (Hochman, 1997). In addition, “proapoptosis” was proposed to be involved in population differentiation, such as heterocysts differentiation in *Anabaena* (Hochman, 1997). Shortly after, experimental work and field observations evidenced the induction of autocatalytic cell death processes in response to abiotic stresses (nutrient starvation, high light, osmotic stress, and oxidizing agents) that were designated as “PCD.” Common biochemical and morphological features associated with these pathways include thylakoid disintegration, loss of plasmalemma integrity and vacuolation, cell shrinkage, DNA fragmentation, oxidative stress, and increased protease activities (Ning et al., 2002; Berman-Frank et al., 2004; Ross et al., 2006). The pioneer reports inspired numerous subsequent studies, which were focused on RCD processes induced mainly in bloom-forming species subjected to harsh non-physiological treatments. These led to a significant body of literature describing cell death processes based on variable amounts of evidence and diagnostic markers, which have been named: “PCD,” “PCD-like,” “apoptosis,” “apoptotic-like,” “apoptosis-like PCD,” “autophagic-like,” “necrotic-like,” “autolytic-like,” and “ferroptosis-like” (Figure 1; Table 1; Zheng et al., 2013; Yang et al., 2017; Aguilera et al., 2019; Hu and Rzymski, 2019).

**Table 1:** Summary of the studies performed in RCD markers identified in Cyanobacteria under different triggers.

Population/Cyanobacterial strain	Cell death trigger	Cell death quantification	Cell death designation	Molecular/genetic analysis	Morphologic studies	Biochemical characterization	Antioxidant quantification	ROS production	Lipid peroxidation	Reference
Field populations of *Prochlorococcus* and *Synechococcus*	nd	Enzymatic cell digestion method	Cell death	nd	nd	nd	nd	nd	nd	Agustí and Sánchez, 2002; Agustí, 2004; Llabrés et al., 2011
*Anabaena* sp. 7120, *A. cylindrica*, *A. siamensis, A. flosaquae*	Osmotic stress (salt addition)	Hoechst 33342, DAPI	PCD	nd	TEM	Tunel, DNA ladder	nd	nd	nd	Ning et al., 2002
*Trichodesmium* IMS101, *Trichodesmium erythraeum*	Phosphorus and iron starvation, high irradiance, and oxidative stress	nd	PCD	nd	TEM	Tunel, DNA ladder, caspase activity	nd	nd	nd	Berman-Frank et al., 2004
*Field population of Anabaena flosaquae* and *Microcystis flosaquae*	Fungal infection, senescence	Evans Blue,Hoechst stain	PCD	nd	nd	Tunel	nd	nd	nd	Sigee et al., 2007
Endosymbiotic cyanobacterium of *Azolla microphylla*	Darkness and nutrient deprivation	DAPI,Chlorophyll fluorescence	Apoptotic-like, autophagic-like, autophagic-like, autolytic-like	Identification of caspases genes	TEM	Tunel, annexin V	nd	nd	nd	Zheng et al., 2013
*Anabaena* PCC7120	Temperature (45°C)	DAPI	Apoptosis	RT-PCR	nd	nd	nd	nd	nd	Tiwari et al., 2016
*Synechocystis* (glucose-tolerant strain)	Temperature (50°C)	SYTOX green, chlorophyll	PCD	nd	nd	nd	nd	nd	nd	Srikumar et al., 2017
*Anabaena fertilissima*	Osmotic stress (salt addition)	DAPI	PCD	nd	nd	Annexin V, DNA ladder	Glutathione	DCFH-DA	nd	Swapnil et al., 2017
Field populations of *M. aeruginosa*	nd	SYTOX Green	Cell death	nd	nd	Annexin V	nd	nd	nd	Kozik et al., 2019
*Synechocysts* sp. PCC 6803	Temperature (50°C)	FDA, SYTOX green	Ferroptosis	RT-PCR	TEM	Caspase activity	Glutathione and Ascorbid Acid	H_2_DCFDA	BODIPY	Aguilera, et al. 2019
*T. erythraeum* IMS101	Nutrient depletion and light (Fe depletion, high light, combination of both)	nd	PCD	qRT-PCR	nd	Caspase activity	nd	nd	nd	Spungin et al., 2019
*M. aeruginosa* Dianchi905	H_2_O_2_	SYBR green, PI	Apoptosis-like programmed cell death	Transcriptional analyses	SEM/TEM	Tunel, caspase activity	Glutathione	nd	nd	Zhou et al., 2020
*M. aeruginoa* SAG 14.85	Grazing pressure (Exposure to Daphnia).	nd	PCD	nd	nd	Caspase activity	nd	x	x	Rzymski et al., 2020
*Halothece* sp. PCC 7418 and *Fischerella muscicola* sp. PCC 73103	Nutrients (phosphate and iron)	nd	Apoptosis	Bio-informatic analysis describing the predicted Pho, Fur, and NtcA regulons.	TEM	Annexin V	nd	x	nd	Fernández-Juárez et al., 2020

PCD, Programmed cell death; TUNEL, terminal deoxynucleotidyl transferase dUTP nick end labeling; TEM, transmission electron microscopy; ROS, reactive oxygen species; PI, propidium iodide; DAPI, 4',6-diamidino-2-phenylindole; DCFH-DA/ H_2_DCFDA, 2',7'-dichlorofluorescin diacetate; nd, not determined. A table summarizing previous studies on cell death in Microcystis is found in Hu and Rzymski (2019).

Nevertheless, the term PCD is the most widely used term to refer to all instances of RCD, and is often used interchangeably with apoptosis (Franklin et al., 2006; Bidle, 2015; Hu and Rzymski, 2019; Bhattacharjee and Mishra, 2020). Notably, the same morphological and biochemical features, in particular cytoplasmic vacuolation, DNA fragmentation, and caspase activity, are claimed to be hallmarks of PCD in some studies (He et al., 2016) while hallmarks of apoptosis in others (Ding et al., 2012; Li et al., 2020; Zhou et al., 2020). Of note, caspase activity – also found in the literature as “protease activity,” “caspase-like activity,” “caspase-like proteolytic activity,” or “metacaspase-like activity” – is considered the main diagnostic marker of both “PCD” and “apoptosis” in cyanobacteria (Ning et al., 2002; Berman-Frank et al., 2004; Ross et al., 2006; Ding et al., 2012; Spungin et al., 2019). However, there are still several misconceptions and uncertainties to be solved regarding increased proteolytic activities in this group, which are further discussed in section Proteolytic Events and Cell Death in Cyanobacteria. Lastly, necrosis is used to refer to all passive and non-controlled types of cell death, and often considered a strict equivalent of ACD (Franklin et al., 2006; Nürnberg et al., 2014; Bidle, 2016; Hu and Rzymski, 2019; Zhou et al., 2020). Taken together, this evidence reflects the lack of consensus regarding (i) the amount and type of evidence needed to define major cell death modalities, and (ii) the terminology adopted to refer to cell death types and subroutines in cyanobacteria. Importantly, it also highlights the imperative need for an updated and common classification.

## Cell Dead Quantification in Cyanobacteria

Despite its frequent use, the concept of cell death still remains difficult to define. According to the NCCD guidelines, cell death occurs when the cell loses plasma membrane integrity or undergoes complete cellular fragmentation (Galluzzi et al., 2018). For cyanobacteria, the distinction between life and death is sometimes problematic. In this section, we review the available methods used to determine viability in this group, with a focus on those based on the loss of membrane integrity that characterizes dead cells. Of note, cell death examination and quantification has been relatively straightforward in laboratory experiments but less commonly applied in natural populations.

The plate count method is the standard way to determine cell viability in microbiology, and relies on bacteria growing as colonies on a plate that become visible to the naked eye and therefore can be counted. This technique, however, is indirect, determines colony-forming units instead of living cells, and may sub estimate population viability. Further, the plate count method is slow, requiring at least 1 week until colonies can be identified, and has several limitations when used for environmental samples (Jones, 1987; Davey, 2011).

The enzymatic cell digestion method is a non-staining test based on the selective digestion of dead cells by the enzymes: Trypsin and DNAse I. These enzymes lead to peptide hydrolysis and the fragmentation and hydrolysis of DNA, respectively, only in cells with damaged plasma membranes, and have no effect on the viability, morphology, or function of live cells (Agustí and Sánchez, 2002). The resulting cell suspensions only present alive cells and can be examined under light microscopy (Agustí and Sánchez, 2002; Agustí et al., 2006). In contrast to methods employing vital stains (reviewed below), this technique does not involve fluorescence signals that may interfere with natural fluorescence of phytoplankton. The enzymatic cell digestion method was successfully applied to assess viability in cyanobacterial cultures and also in natural phytoplankton communities (Agustí and Sánchez, 2002; Agustí et al., 2006; Llabrés et al., 2011).

When compared, the percentages of living cells obtained using the enzymatic cell digestion method were tightly correlated with the ones obtained using the BacLightTM Viability Kit and applied to freshwater samples from eutrophic lakes dominated by cyanobacteria (Agustí et al., 2006). The BacLightTM Viability Kit employs a dual staining procedure using SYTO 9 (green fluorescent) that penetrates most membranes freely, and propidium iodide (PI; red fluorescent) that is polar and can only enter cells with compromised or damaged membranes (Berney et al., 2007). SYTO 9 and PI were also used together to evaluate cell viability in *Microcystis aeruginosa* PCC 7806 and *Synechocystis* sp. PCC 6803 under fluorescence microscopy (Zhu and Xu, 2013).

A simple dual-fluorescence viability assay using SYTOX Green was tested on several cyanobacteria such as *Synechocystis* sp. PCC 6803, *Synechococcus* sp. PCC 7492, *Anabaena* sp. PCC 7120, *Oscillatoria agardhii* NIES-204 and *Phormidium foveolarum* NIES-32. SYTOX Green has a high affinity to nucleic acid and only penetrates damaged cell membranes and does not overlap with chlorophyll autofluorescence. Therefore, SYTOX Green fluorescence and chlorophyll autofluorescence can be used simultaneously as markers for dead and live cells, respectively (Sato et al., 2004). An alternative method to assess cell viability based on chlorophyll fluorescence without the need of dyes was established and validated for *Synechocystis* sp. PCC 6803 (Schulze et al., 2011). In this assay, red chlorophyll fluorescence and green autofluorescence are used for the differentiation of viable and non-viable cells, respectively (Schulze et al., 2011).

Regarding natural populations, SYTOX Green has been employed to examine cell death in *M. aeruginosa* (Kozik et al., 2019). Evans blue in combination with Hoechst staining have been applied to evaluate the occurrence of senescence and PCD in natural freshwater populations of *Anabaena flos-aquae* and *Microcystis flos-aquae* during summer blooms (Sigee et al., 2007; Table 1).

It is worth mentioning that epifluorescence microscopy may under or overestimate cell viability if not recorded properly and fast enough (Chapman et al., 2016). Flow cytometry stands as a powerful technique to test cell viability and also metabolic assessment for its speed, accuracy, and recording of multiple parameters (Dashkova et al., 2017). PI and fluorescein diacetate (FDA) were used simultaneously to quantify cell viability in *Microcystis* in co-culture with *Bacillus mycoides* B16 using flow cytometry. FDA is cleaved by non-specific intracellular esterases releasing fluorescein that is retained inside metabolically active cells and emits green fluorescence under blue light excitation (Gumbo et al., 2014). DAPI (4,6-diamidino-2-phenylindole) has been used to evaluate cell death and also to report apoptotic events in cyanobacterial studies (Ning et al., 2002; Zheng et al., 2013; Tiwari et al., 2016; Swapnil et al., 2017; Table 1). This blue-fluorescent DNA stain enhances fluorescence upon binding to AT regions of dsDNA and is frequently used in animals to detect apoptosis (Cummings et al., 2004). Taken together, to avoid misinterpretations and reach more accurate results, it is highly recommended to combine more than one assay to assess cell viability, for instance, we suggest the combination of fluorescent microscopy with SYTOX green and flow cytometry with FDA (Aguilera et al., 2019).

## Biochemical and Molecular Aspects of Cell Death

The NCCD formulates periodic and extensive updates to unify the criteria used to define cell death subroutines in metazoans (Kroemer et al., 2009; Galluzzi et al., 2012, 2018). Cell death subroutines in cyanobacteria (reviewed in section Types and Subroutines of Regulated Cell Death in Cyanobacteria) are commonly defined by morphological criteria, without a clear description of the biochemical mechanisms. Therefore, as a first step, it would be essential to clarify and unify criteria regarding cell death types and subroutines in this group. This will allow a better understanding of this topic between authors, reviewers, and editors.

A complete review of the biochemical methods available to study cell death in *Microcystis* was recently published (Hu and Rzymski, 2019). For this reason, we will focus on the biochemical tools employed in other cyanobacteria and in two new studies in *Microcystis*. Several biochemical and morphological tools are employed in studies reporting cell death (Table 1) which have allowed reaching important conclusions for the field of RCD in cyanobacteria but are incomplete to define a cell death subroutine. In addition, expressions such as “percentage of apoptosis” or “percentage of PCD” are frequently used without mentioning the method used. Since such expressions are imprecise, they should be abandoned and replaced by more descriptive ones such as “percent Sytox Green-positive,” “percent annexin V-binding,” “percent active caspase-3-like positive,” and “percent TUNEL positive” cells. Genetic studies employing mutants, functional and pharmacological assays using specific inhibitors are still needed to decipher the pathways of the subroutines operating in cyanobacteria. Importantly, this will allow finding new control points to develop technological tools for the management of cyanobacterial blooms.

The production of reactive oxygen species (ROS) has been often associated with cell death in both animals and plants (Kroemer et al., 2009; Distéfano et al., 2017). Similarly, a sharp increase in ROS production has been reported for different cyanobacteria species upon environmental stress. Interestingly, oxidative stress is induced in *M. aeruginosa* under several triggers such as environmentally disturbing conditions and chemical compounds (Table 1; reviewed by Hu and Rzymski, 2019). *Anabaena fertilissima* exposed to 250 mM NaCl showed a biphasic ROS production (Swapnil et al., 2017). ROS production has also been detected in *Halothece* sp. PCC 7418 and *Fischerella muscicola* sp. PCC 73103 under nutrient limitation (Fernández-Juárez et al., 2020), and in *Synechocystis* sp. PCC6803 when exposed to heat (Aguilera et al., 2019). The data discussed above provide strong evidence for the participation of ROS in the signaling cascade, acting as a potential mediator in signal transduction pathways, as reported for eukaryotes (Fleury et al., 2002; Achard et al., 2008; Newton et al., 2016).

Two standing questions are the source and the type of ROS involved in cyanobacterial cell death. Until now, the probe 2',7'-dichlorodihydrofluorescein diacetate (DCFDA, also known as H_2_DCFDA, DCFH-DA, and DCFH) has been commonly used to detect ROS. DCFDA is a fluorogenic dye that measures hydroxyl, peroxyl, and other ROS activity within the cell, thus cannot be used to indicate specific forms of ROS (Marchesi et al., 1999). As for any photosynthetic organism, ROS in cyanobacteria can be produced by both the respiratory chain and the photosynthetic machinery. However, to which extent each of these contributes to ROS production remains unanswered. Importantly, the identification of the source(s) and types of ROS will potentially open the door towards genetic and/or pharmacological experiments. These will be instrumental in gathering evidence to more strongly support the role of ROS in mediating RCD in cyanobacteria.

Overall, the molecular machinery required for the initiation and execution of RCD in cyanobacteria remains largely elusive and has yet to be identified. Hence, knowledge on the molecular mechanisms is still strongly needed to define the subroutines operating in cyanobacteria. We present here an update centered on the molecular aspects of the process, reviewing the role of the few genes associated with RCD until now.

Metatranscriptomic analysis from the Baltic Sea over two summer seasons suggested stress-response roles for cyanobacterial caspase homologs genes (e.g., sulfur metabolism in connection to oxidative stress; Asplund-Samuelsson et al., 2016). In addition, the co-expression of caspase homologs and cyanotoxin genes suggested a potential relationship between cyanotoxin biosynthesis and cell death. Notably, this study also shows that some of the caspase homologs genes are constitutively expressed in bloom-forming cyanobacteria throughout the whole season, rather than limited to the senescence of the bloom. Thus, caspase homologs genes were speculated to have potential roles in house-keeping functions (Asplund-Samuelsson et al., 2016), as growing evidence supports (Klemenčič and Funk, 2018a).

The *lexA* gene encodes a protein that binds to a palindromic AnLexA-box (AGT-N[4–11]-ACT), regulating about 56 genes in *Anabaena* sp. PCC7120 and *Synechocystis* sp. PCC6803. The regulated genes respond to different abiotic stresses (Kumar et al., 2018). Ssl2245-Sll1130 were identified as a pair of putative heat-responsive Toxin-Antitoxin like transcriptional regulators in *Synechocystis* sp. PCC6803 (Srikumar et al., 2017). Genes involved in glutathione synthesis (*gshA*, *ggt*, and *gpx2*), and iron transporters (*feoB*) were induced after 50°C exposure in *Synechocystis* sp. PCC6803, suggesting that they are involved in the ferroptosis-like signaling pathway (Aguilera et al., 2019).

Whole transcriptome sequencing approaches are a powerful tool to quantify changes in the expression levels of transcripts under different conditions, allowing to reveal new promising candidate genes associated or involved in pathways of interest (Wang et al., 2009). Recently, the transcriptional analysis of *M. aeruginosa* Dianchi905 treated with H_2_O_2_ revealed new genes potentially involved in cell death pathways (“AL PCD”). Genes encoding caspases homologs (identified based on the presence of the peptidase_C14 domain), and those involved in the synthesis of microcystins, energy acquisition and photosynthesis, phosphate utilization, osmotic stress response, DNA repair/SOS response, and *lexA* and *mazE* system were differentially expressed under moderate doses of H_2_O_2_. All these genes seem to be coordinated in activating what authors defined as “apoptosis-like PCD” (Zhou et al., 2020).

## Proteolytic Events and Cell Death in Cyanobacteria

### Caspase-Like Activities: Caspases, Metacaspases, and Orthocaspases

Caspases are cysteine-dependent aspartate-directed proteases, which are present exclusively in animals. Their proteolytic function is enabled by the action of two amino acid residues, His and Cys, situated in a so-called p20 domain. Proteins containing this domain were identified in unicellular eukaryotes (protozoa, yeast, and algae) as well as in unicellular prokaryotes (bacteria and archaea) and were termed metacaspases (Uren et al., 2000) and orthocaspases (Klemenčič et al., 2015), respectively. An important distinction between metacaspases and orthocaspases is the presence of specific aspartate rich motives on the p10 domain in metacaspases. These motives enable binding of calcium ions and thus render such proteases calcium-dependent (Tsiatsiani et al., 2011; Choi and Berges, 2013; Minina et al., 2013). Orthocaspases, on the other hand, lack these motives and are thus calcium-independent proteases. Another feature is the presence of additional domains on the same polypeptide chain, which is observed only in orthocaspases. Many of them mainly contain (but not necessarily do) additional domains C-terminally to the p20 domain (Klemenčič and Funk, 2018a). In cyanobacteria, only two types of caspase homologs are found: type I metacaspases and orthocaspases (Jiang et al., 2010; Asplund-Samuelsson et al., 2012). It should be mentioned that cyanobacteria are especially rich in proteolytically inactive orthocaspase variants (Klemenčič et al., 2019; Lema et al., 2021). In these proteins, the catalytic dyad is substituted with catalytically inactive amino acid residues. Since they cannot perform proteolysis, they will not be further discussed here.

### Measuring Orthocaspase and Metacaspase Activities During RCD

Caspases have been a prominent target for diagnosing cell death in cyanobacteria, given their biochemical role in metazoan RCD (Shalini et al., 2015). Cyanobacteria lack true caspases but contain various structural homologs (metacaspases and orthocaspases; Klemenčič and Funk, 2018a). To date, only one cyanobacterial caspase homolog, an orthocaspase of *M. aeruginosa*, has been experimentally characterized (Klemenčič et al., 2015).

Given the recognized importance of caspases during RCD processes in animals, measurement of proteolytic activities of their homologs is commonly employed also in other organisms. However, initial research was based on a misconception that metacaspases and orthocaspases have caspase-like activities. Therefore, tetrapeptides such as DEVD and YVAD, which are known to detect caspase-like activities in animals, were used to measure the activities of metacaspases and orthocaspases as well. However, this did not provide any evidence of activity and is, in fact, not correct. To this day, all characterized non-metazoan caspase homologs, including ortho-/para-/metacaspases, represent structural homologs, e.g., they share the same protein fold, but have contrasting substrate specificities. As opposed to caspases, metacaspases and orthocaspases cleave their substrates after a positively charged amino acid residue at the P1 position (Vercammen et al., 2004; Bozhkov et al., 2005; He et al., 2008; Watanabe and Lam, 2011; Klemenčič et al., 2015; Klemenčič and Funk, 2018b). While the use of such substrates (e.g., FR-AMC, RR-AMC, VRPR-AMC) is now common for biochemical characterization of recombinantly prepared proteins, many literature reports still wrongly suggest measurement of ortho-/metacaspase activities by using caspase substrates during “PCD” processes in cell culture or environmental samples. Up to now, only two studies employed Ac-VRPR-AMC (Ac-Val-Arg-Pro-Arg-AMC) for monitoring orthocaspase activities (metacaspases, according to the authors) during cell death processes (“PCD,” according to the authors) in both *Trichodesmium* cultures and environmental samples (Spungin et al., 2018, 2019). In both studies, caspase-like activities were monitored concomitantly using the fluorogenic caspase substrate Z-IETD-AFC (Z-Ile-Glu-Thr-Asp-AFC). Results suggest a significant correlation between both orthocaspase-like and caspase-like activities and “PCD” mortality in cultures and in oceanic populations (Spungin et al., 2018, 2019). This observation is rather interesting and implies that either overlap in substrate specificities between caspase-like and orthocaspase-like activities exists, or that cell death processes in cyanobacteria involve a general increase in cellular proteolytic activities. To test the first hypothesis, biochemical activity was monitored in the presence or absence of various inhibitors. These assays have demonstrated that orthocaspase-like and caspase-like activities are likely distinct and are independently activated under stress and coupled to “PCD” in both laboratory and field populations (Spungin et al., 2019). These results would therefore suggest the use of non-specific substrates [azocasein, fluorescein isothiocyanate (FITC)-labeled casein, etc.] to test if the measured increases truly correspond to increases of specific proteolytic activities or are just a consequence of increased overall proteolytic activities of the cell as a response to stress.

### Caspase-Like Activities and RCD

As described above, caspase-like activities are detected in cell death processes in organisms lacking true caspases. In cyanobacteria, “PCD” processes are accompanied with increased caspase-3 like activities, which are usually measured using synthetic peptides such as DEVD (Ross et al., 2006; Bouchard and Purdie, 2011; He et al., 2016; Lu et al., 2017) or IEDT (Bar-Zeev et al., 2013). Despite the lack of true caspases in these organisms, the caspase-like activity seems to be a hallmark of “PCD” processes also in cyanobacteria. Indeed, proteases with caspase-like activity (cleavage of substrates with Asp/Glu residues at P1 position) are becoming increasingly recognized as the key enzymes of plant RCD. Among them are cathepsin B (Ge et al., 2016), vacuolar proteolytic enzyme (VPE; Hatsugai et al., 2004), subtilase (Coffeen and Wolpert, 2004), and phytaspase (Chichkova et al., 2018). Bioinformatic analyses have revealed the presence of genes encoding the above-mentioned serine proteases in cyanobacteria (Tripathi and Sowdhamini, 2008), while genes encoding VPEs seem to be absent. With the exception of cathepsin B in *Leishmania* (El-Fadili et al., 2010; Gerbaba and Gedamu, 2013), no proteases responsible for caspase-like activity have been identified in any unicellular organism, including prokaryotes. Thus, the protein/s responsible for the observed proteolytic activities remain to be also identified in cyanobacteria.

## Types and Subroutines of Regulated Cell Death in Cyanobacteria

### Programmed Cell Death

The term “PCD” has been traditionally employed to refer to all types of non-accidental cellular demise in cyanobacteria. Besides, most of our knowledge comes from studies where cyanobacteria were subjected to non-physiological stress conditions. Thus, one question that remains open is whether cyanobacteria undergo cell death not only when subjected to external environmental perturbations, but also in the context of physiological programs to control development, homeostasis, and differentiation. That would define this type of death as PCD as considered for multicellular organisms by the NCCD (Galluzzi et al., 2015). Given the role of PCD in metazoan development, it is tempting to seek for a similar adaptive role for cell death in cyanobacteria, in particular in the more complex forms. The potential role of PCD in cyanobacterial population dynamics is further discussed by Franklin (2021, this Special Issue).

Filamentous forms possess several hallmark traits reminiscent of complex eukaryotic multicellularity (Herrero et al., 2016) and show levels of reversible or terminal cell differentiation. The most complex filamentous species differentiate up to five different cell types: vegetative cells, heterocysts, akinetes, hormogonia, and necridia (Claessen et al., 2014; Herrero et al., 2016). In these forms in which differentiation occurs particularly to facilitate dispersal, cell death could have a programmed dimension controlled by intercellular signaling. Below we present the few available observations that might denote such instances.

Filamentous forms of the order Nostocales differentiate heterocysts when a combined nitrogen source (such as nitrate or ammonia) decreases or is removed. These cells provide a microoxic environment necessary for the production and proper functioning of nitrogenase and other proteins related to atmospheric nitrogen fixation (Flores and Herrero, 2010). The terminal differentiation of heterocysts in *Nostoc punctiforme* has been proposed as a basic form of “PCD” or “apoptosis” since the cellular consequences would be identical to those observed in eukaryotes (Meeks et al., 2001).

Akinetes and hormogonia have an important role in the persistence and dispersal of filamentous cyanobacteria (Meeks et al., 2002; Kaplan-Levy et al., 2010). However, the role of cell death in propagule formation is largely unrecognized in this group. Interestingly, both akinetes and hormogonia are thought to be released in the environment as a result of “PCD” processes, even though the precise experimental proof is still required (Claessen et al., 2014). Akinetes are spore-like cells that differentiate from vegetative cells, acting as resting stages and also serving as dispersal units (Kaplan-Levy et al., 2010). Even though factors triggering akinete differentiation vary between different species, the most important are light quality and intensity, temperature, and nutrient limitation (Kaplan-Levy et al., 2010). Hormogonia are short motile or immotile trichomes that are released from the parental trichome in several filamentous heterocyst-forming and non-heterocyst-forming cyanobacteria (de Marsac, 1994; Meeks et al., 2002). Numerous environmental factors, including light and nutrients, can stimulate or inhibit hormogonium differentiation and its release from the parental trichome is mediated by necrotic processes leading to the death of several cells, called necridia. Necridia differentiation was then considered as a basic form of “PCD” (“apoptosis”; Nürnberg et al., 2014).

Dispersal has also been proposed to be the adaptive basis for the death of specific cells leading to trichome fragmentation in *Calothrix elenkinii*. In this cyanobacterium an alternative process of fragmentation without the involvement of necridia was reported under green light and nitrate starvation. The separation of the trichome into two fragments of similar length is mediated by the death and disintegration of one cell near its center which displays high chlorophyll fluorescence followed by the abrupt decomposition of the photosynthetic apparatus. This is followed by cell bleaching and shrinking which leads to its ultimate death within a few minutes. This process continued repetitively until the parental trichome breaks up into short nonmotile fragments of equal length and therefore considered a case of “PCD” (Adamec et al., 2005).

Finally, a detailed morphological and biochemical analysis revealed the existence of several modes of cell death in a filamentous cyanobacteria endosymbiont of the fern *Azolla microphylla*. The authors described “apoptotic-like,” “autophagic-like,” “autolytic-like,” and “necrosis-like” cell death based on different sets of ultrastructural and biochemical features (Zheng et al., 2013). Cell death modes were related with the developmental stage of the host plant. For instance, “apoptotic”- and “autophagic-like” morphotypes predominantly occurred in the early developmental stage of the host plant (young plant parts) while necrotic- and autolytic-like events were evident in organisms placed in older leaves. “Apoptotic-like” cell death was characterized by externalization of phosphatidylserine detected by annexin V, cell shrinkage, and thylakoid disintegration. Cells undergoing “apoptotic-like” presented cellular inclusions such as cyanophycean granules and carboxysomes and maintained intact the outer membrane. Cells did not form apoptotic bodies, but presented a highly irregular dendritic shape. The externalization of phosphatidylserine was also evident in cells undergoing “autophagic-like” cell death, together with cytoplasmic disintegration and a high level of vacuolation. “Necrotic-like” cell death was characterized by cell wall rupture at an early stage and the leakage of cellular content. Lastly, cell wall lysed at an early stage during the “autolytic-like” mode, while cells showed cytoplasmic disintegration and the formation of vesicles. All cell death modes were observed in vegetative cells but also in heterocysts and akinetes, potentially linking cell death with cell differentiation. In addition, *Azolla* plants were subjected to external abiotic stresses (darkness, nutrient deprivation, and gamma radiation). Interestingly, the treatments further enhanced cell death in the cyanobacteria in a dose-dependent manner (Zheng et al., 2013).

Overall, detailed experimental evidence demonstrating that RCD is linked to cell differentiation is still lacking. Whether RCD pathways are involved in the differentiation of specific cells into heterocysts, akinetes, or the death of the vegetative cells surrounding the akinete, remains to be elucidated. Experimental proof is still required to better understand the connection between cell death pathways and the differentiation of hormogonia and necridia. On the other hand, it is also arguable whether these instances of cell differentiation indeed represent cases of PCD (*sensu* the NCCD) since they are induced or enhanced by external and non-physiological conditions that are considered stressful for the cells (e.g., low temperature or nutrient deprivation). Consequently, in view of the lack of evidence of cell death processes strictly involved in physiological programs, it is suggested that the use of RCD should be preferred over PCD.

### Apoptosis-Like

The terms “apoptosis,” “apoptotic-like,” “apoptosis-like PCD” have been applied in cyanobacteria for cell death events that occur under abiotic stresses while manifesting several morphological and biochemical features described for metazoan extrinsic apoptosis (Galluzzi et al., 2018). In mammalian cells, this cell death subroutine is initiated by perturbations of the extracellular microenvironment and involves specific plasma membrane death receptors, which are activated with the analog ligands (e.g., FASL/CD95L, BCL-2/BAX, TNF family proteins), dependence receptors, which are activated when levels of their ligands decrease below a specific threshold (i.e., NTN1, NTRK3) and effectors [e.g., death domain (DD), dead-effector domain (DED) proteins]. Besides, it is propagated by caspases (CASP8) and precipitated by executioner caspases, mainly CASP3 (Aravind et al., 1999; Aravind and Koonin, 2002; Galluzzi et al., 2012, 2018). As previously mentioned, typical morphological features observed in mammalian cells undergoing apoptosis are cytoplasmic shrinkage, chromatin condensation, nuclear fragmentation, plasma membrane bebbling, and the formation of apoptotic bodies.

The use of assays that were developed to detect apoptosis in mammals has potentially led to acceptance of apoptosis as a proven concept in cyanobacteria. However, despite several similarities, there also are striking differences. Most of the current knowledge comes from work done in bloom-forming *Microcystis* exposed to abiotic stress conditions such as oxidizing agents (H_2_O_2_), ultra-violet irradiation, herbicides, and allelochemicals (reviewed in Hu and Rzymski, 2019). Furthermore, studies in *A. fertilissima* exposed to high salinity also report “apoptotic-like” events (Swapnil et al., 2017). In general, biochemical features accompanying this type of cell death include the increase in ROS levels, DNA fragmentation, and condensation. Morphological changes include cell shrinkage, cytoplasmic vacuolation, and thylakoid disintegration (Ross et al., 2006; Ding et al., 2012; Swapnil et al., 2017; Zhou et al., 2018, 2020; Ye et al., 2019). Caspase-like activation is considered a conserved hallmark of apoptosis, at least in *Microcystis*, being reported mainly as caspase-3-like activity (Ross et al., 2006; Lu et al., 2017; Zhou et al., 2018, 2020). Recently, a study in *M. aeruginosa* exposed to a range of H_2_O_2_ showed that moderate doses induce “apoptosis-like PCD” (AL PCD), while higher doses lead to non-RCD (necrosis, according to the authors). “AL PCD” is characterized by an early decrease in intracellular ATP content and photosynthetic activity, followed by membrane damage along with an increase of glutathione (GSH) levels, and finally, morphological and structural changes (thylakoid disruption, the appearance of lipid bodies, and cytoplasmic vacuoles). To the best of our knowledge, this is the first study focused on cell death that includes total RNA sequencing (Zhou et al., 2020; further described in section Biochemical and Molecular Aspects of Cell Death).

Overall, the detection of features resembling mammalian extrinsic apoptosis during cyanobacterial cell death does not unequivocally demonstrate its existence. For instance, the specific plasma membrane ligands, receptors, and effectors described for mammals are missing in cyanobacteria. More evidence on the molecular machinery required for the initiation and execution of extrinsic apoptosis is required to conclude whether this cell death subroutine indeed occurs in cyanobacteria. Therefore, it is suggested that the term apoptosis-like should be used over apoptosis, as a way to acknowledge that there are similarities but also distinct differences between the two processes.

### Ferroptosis-Like

Ferroptosis is an iron-dependent, oxidative, non-apoptotic form of regulated necrosis associated with lipid peroxidation and ROS accumulation. This subroutine was initially described in mammals and recently reported in plants and protozoan parasites (Dixon et al., 2012; Berghe et al., 2014; Distéfano et al., 2017; Bogacz and Krauth-Siegel, 2018; Dangol et al., 2018). Emerging evidence suggests that ferroptosis may also operate in cyanobacteria since a similar process is induced in *Synechocystis* sp. PCC 6803 in response to heat, and therefore referred to as ferroptosis-like. After exposure to high temperature (50°C), cells undergo a cell death pathway that can be suppressed by the canonical ferroptosis inhibitors or by external addition of calcium, GSH, or ascorbic acid (AsA). Neither the specific inhibitors nor the external addition of antioxidants suppressed cell death when cells were exposed to a higher temperature (77°C) or H_2_O_2_ (10 mM), suggesting that both extreme conditions led to ACD. No caspase-like activity, assessed by the CellEvent caspase-3/7 green detection reagent, was detected in 50°C-treated cells. Moreover, as described for ferroptosis in eukaryotic cells, the pathway in *Synechocystis* sp. PCC 6803 is characterized by lipid peroxidation and early depletion of the antioxidants GSH and AsA (Aguilera et al., 2019). Thus, GSH depletion could be a potential hallmark to distinguish ferroptosis-like from other types of cell deaths (Zhou et al., 2020). Still, there are additional components of the pathway involved in ferroptosis-like yet to be identified. For instance, even though genes related to glutathione synthesis and iron transport are induced after 50°C exposure (see below), the regulatory networks and the underlying molecular mechanisms are still not fully established in cyanobacteria, and are therefore key subjects of future studies.

### Cell Death Triggered by Phages

In general, cell death is a common fate following viral infection and it may contribute to the release of novel virions or, on the contrary, adversely affect viral replication. In eukaryotic cells, both of animal or plant origin, viral infections can result in apoptosis or regulated necrosis (Jorgensen et al., 2017). Bacterial cell death has been reported upon viral infection and may have primarily evolved as an antiviral defense mechanism (Gao et al., 2019). In biological evolution, viral-like entities likely played a significant role as cell stressors and may even have preceded the origin of cells, as suggested by the primordial virus world scenario (Koonin, 2009). Interestingly, studies in non-photosynthetic bacteria indicate that some genes involved in RCD (e.g., *cid* and *lrg* in *Staphylococcus aureus*) may have viral origin, implying that bacteriophages may have equipped bacteria with these to control cellular infections *via* expression of these genes (Ranjit et al., 2011). The RCD as an antiviral defense system may appear counterintuitive if considered on the level of the single unicellular entity. It does, however, suit the kin selection strategy (Michod, 1982), in which the RCD prevents further lytic propagation of phages and death of other highly genetically alike bacteria, and by that benefits the population and the survival of genes involved in RCD (Hazan and Engelberg-Kulka, 2004; Nedelcu et al., 2011).

The cyanophages are viruses that specifically infect cyanobacteria with a similar life cycle to that of bacteriophages. These consist of adsorption, replication, formation, and dissemination of progeny phages following the lysis of the host cell (Xia et al., 2013). They belong to three families of double-stranded DNA viruses: Myoviridae (with a long contractile tail), Styloviridae (long non-contractile tail), and Podoviridae (short non-contractile tail). However, the state of knowledge in this regard, as well as the information on cyanophage’s abundance and distribution, requires further exploration, e.g., through quantitative PCR (Adriaenssens and Cowan, 2014; Mruwat et al., 2020). Nevertheless, they undoubtedly constitute a significant agent controlling the composition and dynamics of cyanobacterial communities (Proctor and Fuhrman, 1990; Suttle et al., 1990; Mühling et al., 2005). Therefore, their use has also been suggested as a potential method to control the growth of bloom-forming cyanobacteria (Deng and Hayes, 2008). However, in-field observations of *M. aeruginosa* dynamics under cyanophage pressure suggest that they can affect only a small part of the population (Yoshida et al., 2008). This may plausibly be due to genetic diversity in *M. aeruginosa* and shifts between phage-sensitive and phage-insensitive strains. This would need further confirmation. At the moment, it cannot be excluded that RCD plays a protective role in this regard. Moreover, it can be further hypothesized that phage-sensitive and phage-insensitive cyanobacteria may show differences in expression or presence of RCD-mediating factors.

So far, the potentially protective role of RCD during viral infections has not been subject to study. It has been shown that type I and type III CRISPR-Cas systems are widespread in cyanobacteria genomes (Cai et al., 2013; Yang et al., 2015), although experiments in *M. aeruginosa* demonstrated that they play a limited role in defense during the infection process (Wang et al., 2019). Other defense mechanisms against cyanophages include physical barriers such as natural competence and the exopolysaccharide layer (Stucken et al., 2013). Interestingly, recent observations indicate the correlation between RCD and bound exopolysaccharide levels in *M. aeruginosa* cultures exposed to unfavorable conditions inducing oxidative stress (Rzymski et al., 2020). Although in this species exopolysaccharides are known to play a role in colony formation (Gan et al., 2012), they may also provide additional advantages such as protection from viral infection. Moreover, they are produced by the non-colony forming single-filament species, e.g., *Limnothrix* sp. and *Planktothrix agardhii*, which can also be a target of cyanophages (Stucken et al., 2013; Pannard et al., 2016). The kin selection scenario in this regard implies the quorum sensing in which the cells undergoing RCD are releasing signaling molecules to non-infected cells – whether such molecules exist is unknown. It has been however shown that extracellular microcystin promotes the expression of genes involved in exopolysaccharide synthesis in *M. aeruginosa* and that RCD in this cyanobacterium correlates with microcystin release, together suggesting that this metabolite may play a signaling role (Gan et al., 2012; Rzymski et al., 2020). All in all, it remains of particular interest to investigate whether RCD may be induced by cyanophages and if it contributes to cyanobacterial fitness.

## RCD and Harmful Cyanobacterial Blooms

Nowadays, cyanobacterial blooms pose a serious threat to aquatic environments and public health, and are increasing in frequency magnitude and duration globally (Huisman et al., 2018). At present, there is substantial knowledge of the environmental conditions promoting cyanobacterial blooms (see for example Global expansion of harmful cyanobacterial blooms: Diversity, ecology, causes, and controls; special issue of Harmful Algae, vol 54, 2016). In contrast, relatively little is known on how cyanobacterial blooms disappear and which factors trigger and regulate their collapse. The lack of mechanistic understanding of bloom decay processes severely limits the ability to understand, model, and predict their duration and geographic extension.

Once established, blooms can collapse or disperse by physical factors such as cooler temperatures, water column destratification, and increased wind velocities (Paerl and Otten, 2013). Key biotic factors involved in bloom demise include predation by grazers or exogenous infection by fungi or virus (Tijdens et al., 2008; Paerl and Otten, 2013; Gerphagnon et al., 2015). However, as already discussed, over the past two decades it has become clear that cyanobacteria can also undergo RCD (PCD in the literature) in response to environmental stress. As already mentioned, *Microcystis* is the most studied cyanobacteria under laboratory conditions. To date, at least ten types of abiotic stresses have been reported to trigger “PCD” in this cyanobacterium, including oxidants (H_2_O_2_), ultra-violet irradiation, high salinity, herbicides, and allelochemicals (see section Nomenclature and Terms Adopted in the Literature to Refer to Cell Death in Cyanobacteria; reviewed in Hu and Rzymski, 2019). “PCD” was suggested to be involved in the senescence of *Microcystis* blooms in an early study using TUNEL assay to detect the DNA fragmentation in samples collected at the late summer bloom (Sigee et al., 2007). The most extensive work on RCD (PCD, according to the authors) combining laboratory and in-field work has been done in *Trichodesmium* spp., known to form blooms in oligotrophic tropical oceans. In this cyanobacteria, nutrient starvation (iron and phosphorus) and oxidative stress associated with high light induce “PCD” leading to bloom demise mediated by caspase-like proteases (Berman-Frank et al., 2004; Spungin et al., 2018, 2019).

Substantial cell death by lysis has been documented in field populations of marine *Prochlorococcus* and *Synechococcus*. These two picocyanobacteria are not considered as harmful but their ecological importance is remarkable since they are the dominant primary producers in marine ecosystems (Flombaum et al., 2013). The enzymatic cell digestion method (section Cell Dead Quantification in Cyanobacteria) revealed high mortality in *Synechococcus* and *Prochlorococcus* across the central Atlantic Ocean, and the Mediterranean sea (Agustí and Sánchez, 2002; Agustí, 2004; Llabrés et al., 2011). Some estimates exceeded 70% for both genera, evidenced contrasting vertical and spatial distributions, and also clear patterns of diel variation in cell mortality in both cyanobacteria. We still lack, however, detailed information on factors driving cell death and the potential RCD mechanism operating in these cyanobacteria.

All these studies have greatly contributed to our understanding of the ecology of cyanobacteria. Nevertheless, the still limited number of field studies examining cell death in either marine or freshwater systems severely limits the understanding of the environmental conditions that elicit RCD in cyanobacteria. In combination with laboratory assays, field studies are strongly needed to better understand the life cycles of harmful species. Knowledge generated on the triggers and molecular mechanisms of RCD in bloom-forming species represents the basis for the development of specific technological tools (physical treatment or chemical agents, yet to be identified) to prevent and control their massive developments, as a step towards improving public health.

## Recommendations and Future Directions

In the next section, we would like to suggest a few recommendations that we believe will ease communication within the cell death community in cyanobacteria.

The term RCD should be preferred to refer to all instances of cell death involving signaling cascades and intracellular molecular machinery.

It is strongly recommended that the term “PCD” is applied only after the demonstration of its involvement in physiological programs. Studies aiming to better understand the biology of death in cyanobacteria will contribute to disentangle the role of cell death in cell differentiation and will potentially help identify whether there are (or not) PCD pathways in this group.

As recommended for animals and yeast (Carmona-Gutierrez et al., 2018; Galluzzi et al., 2018), the term ACD should be used over necrosis when referring to a type of death caused by extreme conditions or stimuli.

To precisely define cell death subroutines, it is necessary to use biochemical, genetic, and pharmacological approaches rather than morphology. This will allow reaching more accurate conclusions. More efforts need to be done to increase cell death signaling pathways comprehension. In particular, they should aim at identifying the main effectors, membrane ligands, adaptors, second messengers, dead-effector, and executioners of the different subroutines. In addition, pharmacological studies using inhibitors or enhancers of different death subroutines are needed to find tunable points in the death mechanism. If these effectors are specific for cyanobacteria, they could be the principal target to modulate RCD, and therefore potentially used to develop new tools for the management of toxic cyanobacterial blooms without affecting other organisms.

Morphological and ultrastructural inspections (e.g., using transmission electron microscopy) are encouraged to be done in time series to better describe the morphology associated with the studied subroutine.

As explained above, the relationship between proteolytic events and RCD in cyanobacteria can yet not be unambiguously defined. According to the available data, orthocaspase-like as well as caspase-like proteolytic activities are present in populations undergoing RCD. However, two main questions remain unanswered: (i) is RCD in cyanobacteria accompanied by an overall proteolytic activity increase and most importantly (ii) is RCD in cyanobacteria dependent on the proteolytic events as is the case in animals? To provide answers to these questions, we suggest: (i) monitoring of total proteolytic activity using nonspecific-substrates (e.g., FITC-labeled casein) in addition to protease-specific one during RCD (as performed by Spungin et al., 2018, 2019) (ii) using control experiments in which specific inhibitors, targeting the protease in question, are used and the phenotypes of cells with the inhibitor and without it are compared. Additionally, research should be aimed at the identification of the proteases responsible for the observed increased proteolytic activities. This would not only provide the key information regarding the execution of RCD early evolving organisms, but will even more importantly, set the basis for the re-assessment of common RCD mechanisms in all living beings.

Finally, field studies that reaffirm the physiological relevance of laboratory studies are essential. The understanding of the cell death processes that serve to mediate bloom to-post bloom transitions is key to better comprehend how photosynthetically fixed organic matter (and associated elements) flows through the main ecosystem pathways (the grazer food web, vertical sinking flux, and the microbial loop). Furthermore, the comprehension of the mechanisms controlling bloom demise will shed light on the underexplored field of RCD and will importantly open new applications in biotechnology. In particular, in the management of water bodies affected by toxic cyanobacterial blooms.

## Author Contributions

AA and MVM conceptualized the study. All authors contributed to the article and approved the submitted version.

### Conflict of Interest

The authors declare that the research was conducted in the absence of any commercial or financial relationships that could be construed as a potential conflict of interest.
